# Transnuclear CD8 T cells specific for the immunodominant epitope Gra6 lower acute‐phase *Toxoplasma gondii* burden

**DOI:** 10.1111/imm.12643

**Published:** 2016-08-17

**Authors:** Anna Sanecka, Nagisa Yoshida, Stephanie K. Dougan, John Jackson, Nilabh Shastri, Hidde Ploegh, Nicolas Blanchard, Eva‐Maria Frickel

**Affiliations:** ^1^ Host–Toxoplasma Interaction LaboratoryThe Francis Crick InstituteMill Hill LaboratoryLondonUK; ^2^ Whitehead Institute for Biomedical Research and Massachusetts Institute of TechnologyCambridgeMAUSA; ^3^ Division of Immunology and PathogenesisDepartment of Molecular and Cell BiologyUniversity of CaliforniaBerkeleyCAUSA; ^4^ INSERM UMR1043CNRS UMR5282Université de Toulouse‐UPSCentre de Physiopathologie de Toulouse Purpan (CPTP)ToulouseFrance

**Keywords:** antigens/peptides/epitopes, parasitic protozoan, T cells

## Abstract

We generated a CD8 T‐cell receptor (TCR) transnuclear (TN) mouse specific to the L^d^‐restricted immunodominant epitope of GRA6 from *Toxoplasma gondii* as a source of cells to facilitate further investigation into the CD8 T‐cell‐mediated response against this pathogen. The TN T cells bound L^d^‐Gra6 tetramer and proliferated upon unspecific and peptide‐specific stimulation. The TCR beta sequence of the Gra6‐specific TN CD8 T cells is identical in its V‐ and J‐region to the TCR‐*β* harboured by a hybridoma line generated in response to Gra6 peptide. Adoptively transferred Gra6 TN CD8 T cells proliferated upon *Toxoplasma* infection *in vivo* and exhibited an activated phenotype similar to host CD8 T cells specific to Gra6. The brain of *Toxoplasma*‐infected mice carried Gra6 TN cells already at day 8 post‐infection. Both Gra6 TN mice as well as adoptively transferred Gra6 TN cells were able to significantly reduce the parasite burden in the acute phase of *Toxoplasma* infection. Overall, the Gra6 TN mouse represents a functional tool to study the protective and immunodominant specific CD8 T‐cell response to *Toxoplasma* in both the acute and the chronic phases of infection.

## Introduction

*Toxoplasma gondii* is an intracellular protozoan parasite infecting approximately 30% of the global human population. *Toxoplasma* has a wide range of warm‐blooded hosts and in humans can cause disease in immunocompromised individuals and congenital defects in fetuses. A robust T‐cell response mounted in immunocompetent hosts controls parasite growth during both the acute and chronic phases of infection through the production of interferon‐*γ* (IFN‐*γ*).^1^ CD8 T cells have long been known to elicit this critical protective function.^2^, ^3^ Once infected, the parasite persists within cysts in the brain and muscle tissue of the host for its entire life. Crucially, patients with T‐cell deficiencies such as HIV‐infected individuals are highly susceptible to toxoplasmosis.^4^

To study the precise nature of the T‐cell response to an infection it is imperative to identify MHC ligands (or epitopes) recognized by the T‐cell receptor (TCR). We have previously identified epitopes derived from *Toxoplasma* presented on either MHC L^d^ or K^b^ to CD8 T cells.^5^, ^6^, ^7^ We further exploited somatic cell nuclear transfer to create transnuclear (TN) TCR mice specific for two L^d^‐restricted epitopes and one K^b^‐restricted epitope.^8^ All *Toxoplasma* TN mice generated were shown to be functional in their ability to respond to cognate peptide and the K^b^‐restricted TN CD8 T cells were demonstrated to be able to lower parasite load upon transfer to a *Toxoplasma*‐infected animal.^8^

It had long been appreciated that BALB/c (H‐2d) mice compared with C57BL/6 (H‐2b) mice are more resistant to *Toxoplasma* infection and that this trait can be mapped to the MHC I L^d^ locus^9^, ^10^, ^11^, ^12^, ^13^, ^14^, ^15^ and is critically dependent on the parasite strain.^16^ The identification of the HF10 decapeptide derived from the *Toxoplasma* protein GRA6 and the finding that this response is immunodominant explained these earlier observations.^5^ HF10 has an unusual length of 10 amino acids rather than the classic nine amino acids commonly found in H‐2L^d^ MHC I molecules. Moreover, HF10 is polymorphic between different *Toxoplasma* strains, with only type II parasites harbouring the correct epitope.^5^ Interestingly, the C‐terminal location of the HF10 peptide within Gra6 determines its immunogenicity, rather than its affinity for the MHC I molecule or the frequency of the T‐cell precursors.^17^

Here, we report the TN CD8 T‐cell mouse specific for the Gra6 immunodominant epitope. We show that the antigen‐specific CD8 T cells from this mouse are responsive to cognate peptide and functional. We further established that Gra6‐specific TN CD8 T cells are efficient at reducing the parasite load in infected mice, and that Gra6 TN mice themselves are more resistant to *Toxoplasma* infective burden. Upon sequencing of the TN TCR from the Gra6‐specific mouse we found that the *β*‐chain is shared with the *β*‐chain of the TCR expressed by a traditionally generated hybridoma responsive to Gra6 peptide.

## Materials and methods

#### Mice

Transnuclear mice were generated as previously described.^8^, ^18^ Briefly, 2 weeks after intraperitoneal infection with *T. gondii* Pru tachyzoites, splenic CD8^+^ Gra6 tetramer^+^ cells were sorted by FACS and used as a source of donor nuclei for somatic cell nuclear transfer (SCNT). The mitotic spindle was removed from mouse oocytes and replaced with donor nuclei. SCNT blastocysts were used to derive embryonic stem cell lines. These embryonic stem cell lines were injected into wild‐type B6 × BALB/c F_1_ blastocysts and implanted into pseudopregnant females. The resulting chimeric pups were mated to BALB/c females to establish the Gra6 TN line. All animals used were backcrossed 10 generations onto the BALB/c background. Parkes, Thy1.1 (BALB/c; CD90.1^+^) and TN Gra6 mice on a Rag2‐proficient BALB/c (Rag2^+/+^ CD90.2^+^) background were housed and bred in the animal facility of the Francis Crick Institute (Mill Hill Laboratory, London, UK). All experiments were performed in accordance with the Animals (Scientific Procedures) Act 1986.

#### Reagents

Fluorescently labelled antibodies against CD3ε, CD4, CD90.2, CD62L, PD1 and KLRG1 antigens were purchased from Biolegend (San Diego, CA). Fluorescently labelled antibodies against CD8*α* (5H10) and CD69 were purchased from Life Technologies (Carlsbad, CA). H‐2L^d^ monomers with HF10 (HPGSVNEFDF) or photo‐cleavable peptide [YPNVNI(Apn)NF] were obtained from the NIH Tetramer Core Facility (Emory University, Atlanta, GA) and were tetramerized and peptide‐exchanged as described previously.^19^ All peptides were synthesized by Pepceuticals (Leicestershire, UK).

#### Parasites and cells

*Toxoplasma* Pru and CEP tachyzoites were grown in confluent human foreskin fibroblasts maintained in Dulbecco's modified Eagle's medium, 10% fetal calf serum. *Toxoplasma* ME49 (type II) cysts were maintained in the brains of Parkes mice.

#### TCR sequencing

Splenocytes from Gra6 TN mice were washed twice in PBS and CD8 T cells were negatively selected by MACS purification (Miltenyi Biotec, Bergisch Gladbach, Germany). RNA was isolated and 5′‐rapid amplification of cDNA ends (RACE) was performed according to the manufacturer's protocol (Invitrogen, Carlsbad, CA) using reported primers.^20^

#### Genotyping of Gra6 TN mice

Primer trios were designed to detect in one PCR both wild‐type and rearranged Gra6‐specific *α*‐ and *β*‐chains: *α*1: ACATGCGTCCTGACACCTGCTC, *α*2: ACTCACCGGGGCTCTGGCTC, *α*3: AGCATGTGGGGTCAACGGCA, *β*1: GCCCTGCATGCCCCACAGAG, *β*2: TCCGTTCCCAAGCCAAAAGTGGT, *β*3: TCCCACCTGTATGGCCTCTGCC.

#### *In vitro* proliferation assay

Negatively selected CD8 T cells (Miltenyi Biotec) were isolated from spleens and lymph nodes of Gra6 TN mice or wild‐type (WT) BALB/c mice, labelled with 2·5 μm CFSE (Life Technologies) for 5 min at room temperature and stimulated in complete RPMI in 96‐well flat‐bottom plates for 3 days in the conditions described below. For anti‐CD3/28 stimulation, plate‐bound anti‐CD3 (clone 17A2) and anti‐CD28 (clone 37.51) at 5 μg/ml in the presence of 10 ng/ml recombinant mouse interleukin‐2 were employed. For splenocyte stimulation, splenocytes from a WT BALB/c mouse loaded with Gra6 peptide (10 μm) were plated together with CD8 T cells at a 3 : 1 ratio.

#### Adoptive transfer experiments

Lymph nodes from Gra6 TN donor mice were harvested and the released cells were negatively selected for CD8 T cells (Miltenyi Biotec). Recipient Thy1.1 (BALB/c) mice received 10^6^ Gra6^+^ CD8 T cells through intravenous injection before infection. Mice were infected orally with five cysts of the ME49 *Toxoplasma* strain. Cells were harvested at the indicated time‐points during the acute and chronic phases of infection and were processed accordingly.

#### Flow cytometric analysis of spleen, lymph node and brain

Spleens and lymph nodes were dissociated and red blood cells from spleens were removed using red blood cell lysis buffer (Sigma, St Louis, MO). Cells were counted and stained using a panel of antibodies indicated in the experiment. Brain mononuclear cells were isolated via Percoll gradient as described.^21^ Briefly, mice were perfused with cold PBS and brains were isolated and homogenized. Brain cell suspension was diluted to 30% with isotonic Percoll solution and layered on top of 70% isotonic Percoll solution. Gradients were spun for 30 min at 500 ***g***, at 18°. The mononuclear cell population was collected from the interphase of the Percoll gradient, washed and resuspended for antibody staining.

#### *In vivo* imaging/parasite load assessment

##### For direct infection

Gra6 TN mice or WT BALB/c mice were infected intraperitoneally with 1 × 10^4^ luciferase‐expressing Pru or CEP *Toxoplasma* tachyzoites.

##### For adoptive transfer experiments

Negatively selected CD8 T cells (Miltenyi Biotec) were isolated from Gra6 TN mice (CD90.2) or WT BALB/c mice. A portion of CD8 T cells from Gra6 TN mice was activated by overnight co‐culture with splenocytes loaded with 10 μm of Gra6 peptide. A total of 1 × 10^6^ cells were injected intravenously into congenic BALB/c (CD90.1) mice. Mice were infected intraperitoneally with 1 × 10^4^ luciferase‐expressing *Toxoplasma* Pru tachyzoites.^22^ An *in vivo* imaging system (Xenogen, Alameda, CA) procedure for imaging *Toxoplasma*‐infected mice has been described in detail previously.^23^ In short, mice were injected with 100 μl of luciferin (3 mg) and immediately anaesthetized. Mice were maintained for 10 min to allow for adequate dissemination of luciferin. Images were collected with 15‐second to 2‐min integration times depending on the intensity of the bioluminescent signal. Data acquisition and analysis were performed using livingimage (Xenogen) software with the igorpro image analysis package (WaveMetrics, Seattle, WA).

##### Statistical analysis

The fluorescence intensity in the parasite load experiment was analysed using two‐way analysis of variance statistical analysis (graphpad prism version 6.0b for Mac OS X GraphPad Software, La Jolla, CA).

## Results

### Somatic cell nuclear transfer of Gra6^+^ CD8^+^ T cells yields a functional Gra6‐specific TN mouse

**Figure 1:**
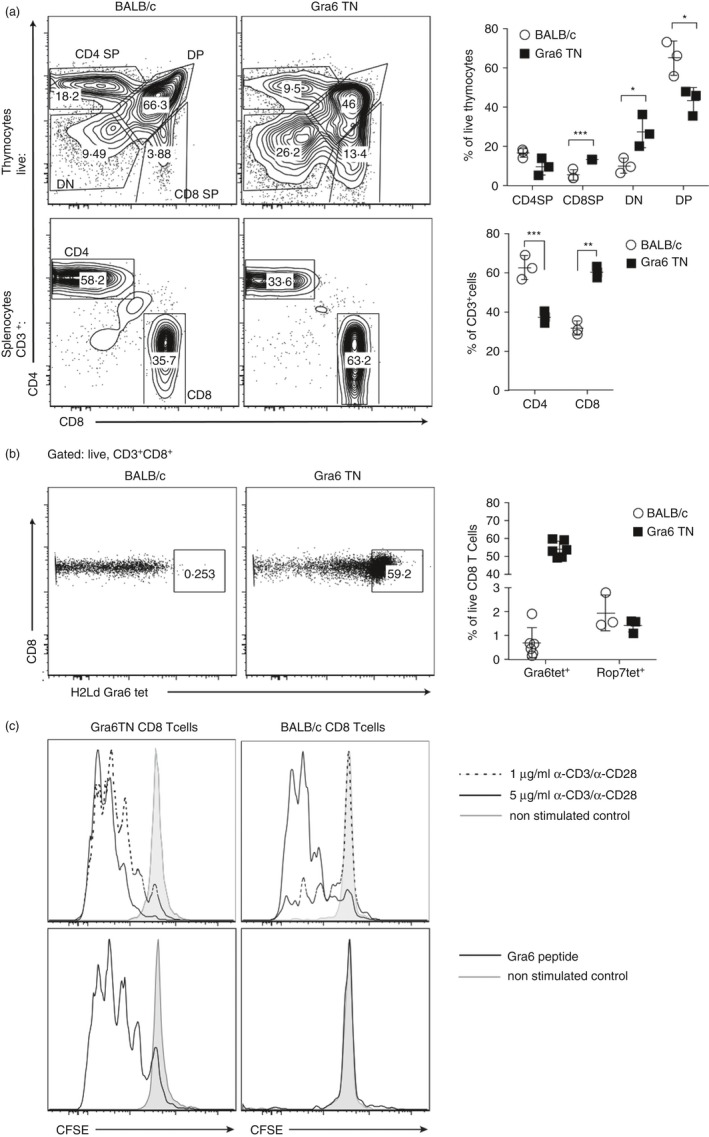
Transnuclear (TN) Gra6 CD8 T cells are functional in response to peptide *ex vivo*. Cells were released from thymus and spleen of wild‐type (WT) BALB/c and Gra6 TN mice and stained for indicated surface markers. (a) Contour plots (left) and graphs (right) show ratios of CD8 to CD4 T cells in thymus (top panel) and in the spleen (bottom panel). Line indicates mean with SD. Statistical significance was calculated with Student's *t*‐test. , * p<0.05, ** p<0.01, *** p<0.001 (b) Splenocytes were stained to show Gra6‐L^d^ tetramer binding on CD8 T cells. The graph on the right shows percentage of the Gra6tet^+^ and Rop7tet^+^ (control tetramer) cells in Gra6 TN and BALB/c mice from three to five mice. Line indicates mean with SD. (c) CD8 T cells from Gra6 TN mice and BALB/c control mice were stimulated with the indicated concentration of plate‐bound *α*‐CD3/*α*‐CD28 (upper panel) or Gra6 peptide‐loaded splenocytes (lower panel). Shaded histograms represent non‐stimulated control.

We sought to produce a CD8 TCR mouse specific to the immunodominant epitope derived from the dense granule protein GRA6 from *T. gondii* in order to further study the nature of this response. Gra6‐specific CD8 T cells were sorted from *Toxoplasma*‐infected spleens of BALB/c × C57BL/6 F_1_ mice using Gra6‐loaded H2‐L^d^ tetramers, and TN mice were generated as described previously.^8^ Chimeric mice were backcrossed to BALB/c mice to achieve germline transmission and subsequently further backcrossed for at least 10 generations. We compared the lymphocytes in thymus and spleen of Gra6 TN mice with those of WT BALB/c mice (Fig. 1a). As expected, we observed an increased percentage of single‐positive CD8 thymocytes as the prearranged Gra6‐specific TCR is already determined before the development of CD8 T cells. Similarly to the thymus, in the spleen we also observed a reversed ratio of CD4 and CD8 T cells between WT BALB/c and Gra6 TN mice. An additional difference, a decreased percentage of double‐positive thymocytes and an increased percentage of double‐negative thymocytes (Fig. 1a) was previously attributed to lymphocytes in TN mice as a sign of smooth passage through negative and positive selection and earlier expression of prearranged TCR genes.^8^

Gra6 TN mice exhibited about 60% of Gra6‐specific CD8 T cells in their splenic population as assessed by binding to H2‐L^d^ tetramer loaded with Gra6 peptide (Fig. 1b). As both the Gra6 TN mice and BALB/c mice showed < 2% tetramer‐positive staining with the unrelated Rop7‐Ld tetramer, we considered this to be background staining. BALB/c mice contain < 1% background levels of Gra6‐specific CD8 T cells (Fig. 1b). Upon unspecific stimulation with anti‐CD3 and anti‐CD28, Gra6 TN cells proliferated as well as upon stimulation with splenocytes loaded with Gra6 peptide (Fig. 1c). We therefore concluded that we had generated a Gra6‐specific TN mouse model that is suitable to further investigate this specific immunodominant CD8 T‐cell response to *Toxoplasma*.

### Identification of the TCR sequence in CD8 T cells specific for the Gra6 epitope

**Figure 2:**
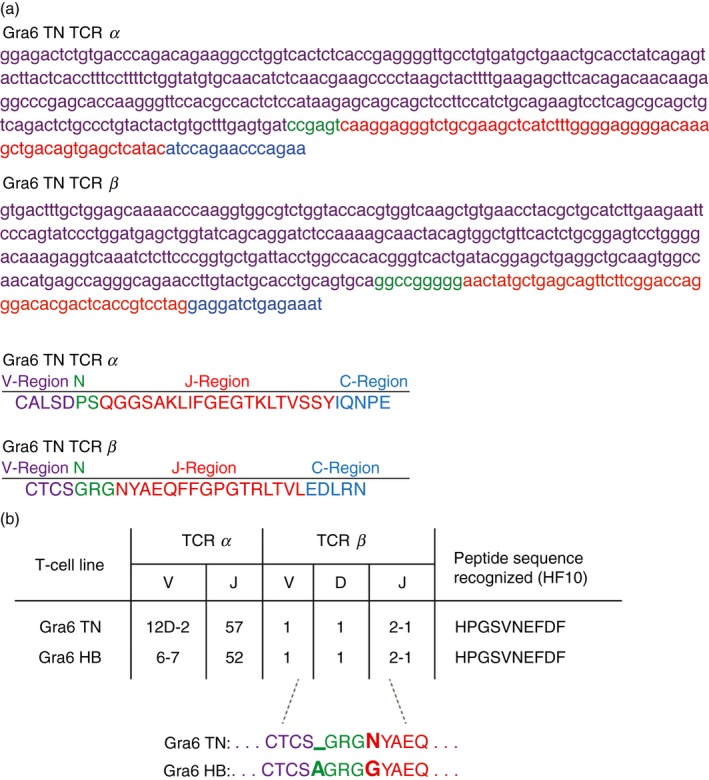
T‐cell receptor (TCR) sequence of Gra6 transnuclear (TN) T cells and transgenic (TG) hybridomas resemble each other. (a) Nucleic acid sequences of the *α* and *β* chain of the Gra6 TN TCR and amino acid sequence of CDR3 regions of *α* and *β* chain of the Gra6 TN TCR. (b) The table represents a comparison of the gene and allele usage by the *α* (V and J) and *β* (V, D and J) chains of the TCRs and a comparison of the amino acid sequence of CDR3 region of *β* chains of the TCRs of the TN CD8 T cells (Gra6 TN) and a CD8 T‐cell hybridoma (Gra6 HB) specific to Gra6 peptide (HF10).

To be able to track the generated Gra6‐specific TN mice by genotyping, we identified the sequence of the genomic rearranged TCR specific for Gra6 by RACE–PCR. The resulting sequence of the *α*‐ and *β*‐chain of the TCR of the TN mice is depicted in Fig. 2(a). The established genotyping protocol can be found in Materials and methods.

Next, we compared the TCR sequence of the Gra6‐specific TN mice with the sequence of the TCR of an existing *β*‐galactosidase‐inducible reporter CD8 T‐cell hybridoma generated by successive *in vitro* re‐stimulations of splenocytes from *Toxoplasma*‐immunized mice with *Toxoplasma*‐infected macrophages^5^ (Fig. 2b). Different V and J genes are recombined to build the *α*‐chain of the TCR for these two T‐cell clones. Interestingly, for TCR *β*‐chain, the same V, D and J genes were recombined. The sequence of the *β*‐chain of the TCR is almost identical between the two T‐cell clones with the exception of two amino acids in the CDR3 region (Fig. 2b).

### Gra6‐specific TN CD8 cells are responsive to *T. gondii* infection and exhibit an activated phenotype

**Figure 3:**
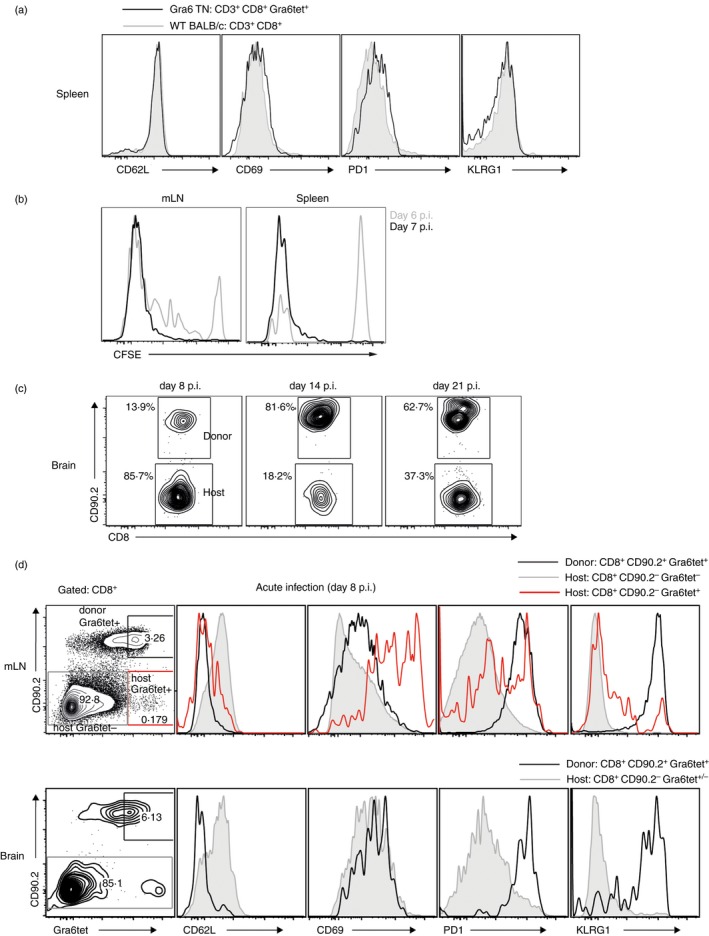
*In vivo* responsiveness of Gra6 transnuclear (TN) CD8 T cells to *Toxoplasma* infection. (a) CD8 T cells from spleens of Gra6 TN mice and wild‐type (WT) BALB/c mice were stained with activation markers. Histograms shown are representative of at least three mice. Cells were gated as live, CD3^+^ CD8^+^ Gra6tet^+^ (black line) for Gra6 TN mice and CD3^+^ CD8^+^ (grey‐shaded histogram) for BALB/c mice. (b, c) Donor CD8 T cells were isolated from spleens and mesenteric lymph nodes (mLN) of Gra6 TN mice using a negative CD8 T‐cell selection kit; 1 × 10^6^ Gra6 TN CD8 T cells were adoptively transferred to congenic mice that were infected orally with five brain cysts of *Toxoplasma* ME49 (type II). (b) Adoptively transferred Gra6 TN CD8 T cells proliferate *in vivo* upon *Toxoplasma* type II infection. Graphs show CFSE dilution of labelled and transferred Gra6 TN cells in the mLN and spleen 6 and 7 days post‐infection with *Toxoplasma*. (c) Gra6 TN cells are detected in the brain at 8 days post‐infection with *Toxoplasma*. Dot plots show the proportion of donor and host CD8 T cells 8, 14 and 21 days post infection in the brain. (d) Phenotype of Gra6‐specific host, adoptively transferred Gra6 TN cells or total host CD8 T‐cell population at day 8 post‐infection in the mLN and brain of *Toxoplasma*‐infected BALB/c recipient mice. One representative plot of three mice is shown per time‐point. In the leftmost contour plots cells are gated as live, CD8^+^. The dot plot on the left indicates the gating strategy for the overlaid histograms: CD8^+^ CD90.2^+^ Gra6tet^+^ (black line), CD8^+^ CD90.2^−^ Gra6tet^−^ (grey‐shaded area), CD8^+^ CD90.2^−^ Gra6tet^+^ (red line).

Gra6 TN CD8 T cells from the spleen exhibit a naive phenotype as shown by low expressions of CD69, PD1 and KLRG1 markers and high expression of CD62L (Fig. 3a). Wild‐type CD8 T cells of BALB/c mice show the same phenotype (Fig. 3a). Active proliferation of adoptively transferred Gra6‐specific TN CD8 T cells was observed *in vivo* between days 6 and 7 post‐infection in the mesenteric lymph nodes (mLN) and the spleen of a congenic BALB/c host infected with *Toxoplasma* (Fig. 3b). When assaying on what day Gra6‐specific CD8 T cells arrive in detectable numbers in the brain of a *Toxoplasma*‐infected BALB/c mouse, we were surprised to encounter host CD8 T cells as well as adoptively transferred Gra6 TN cells as early as 8 days post‐oral infection (Fig. 3c). At day 14, > 80% of CD8 T cells in the brain were donor Gra6 TN CD8 T cells. In the chronic phase of infection (day 21) still most of the CD8 T cells in the brain were from the donor. In the mLN during the acute phase of infection we observed an activated phenotype of donor cells as shown by expression of activation markers: CD62L^lo^, CD69^hi^, PD1^hi^ and KLRG1^hi^ (Fig. 3d). Host Gra6‐specific CD8 T cells were activated in a similar way to donor cells; however, only a small fraction of them was positive for KLRG1. The majority of the host CD8 T cells in mLN at day 8 post‐infection were still in a naive state. In the brain the phenotype of donor cells was also activated and similar to the host population. KLRG1 expression on the Gra6 TN cells in the brain was much higher than on the majority of host cells mirroring the situation in mLN (Fig. 3d). During the course of infection the phenotype of the donor cells did not change drastically. At day 14 and day 21 post‐infection in mLN and in the brain Gra6 TN CD8 T cells exhibited a similar profile to what we observed during the acute phase of infection (see Supplementary material, Fig. S1).

### Gra6‐specific TN CD8 cells confer protection against *T. gondii* challenge

**Figure 4:**
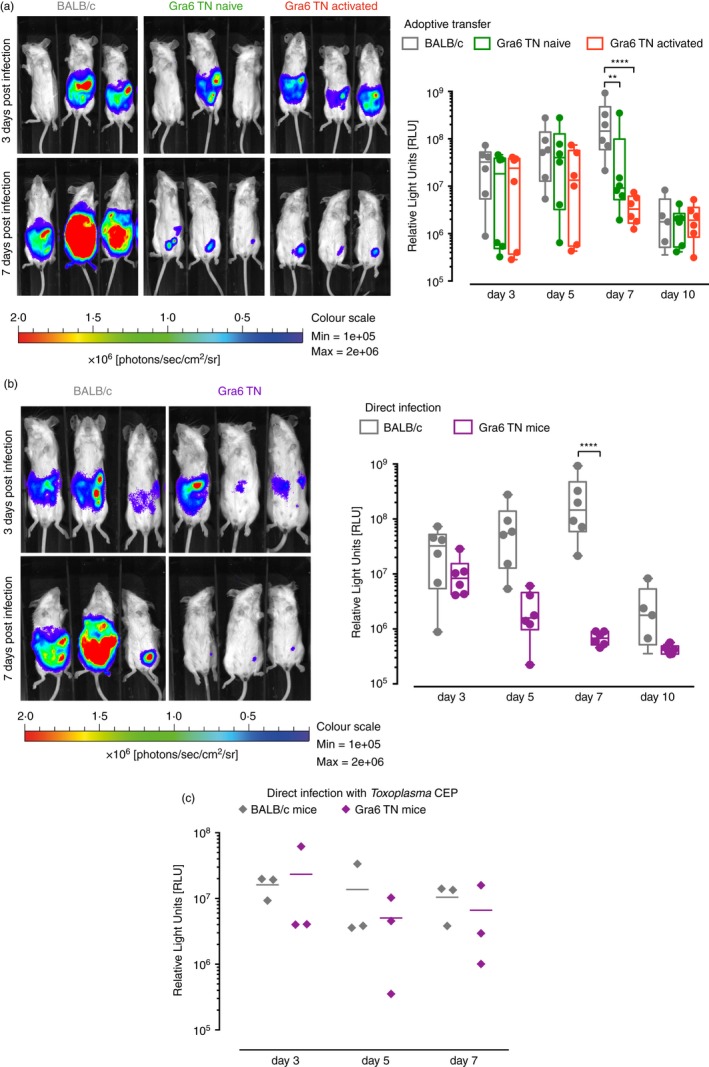
Gra6 transnuclear (TN) CD8 T cells lower the parasite load in *in vivo Toxoplasma* challenge. (a) 1 × 10^6^ Gra6 TN CD8 T cells (naive or activated with Gra6 peptide) or control wild‐type (WT) BALB/c CD8 T cells were adoptively transferred to BALB/c mice. Mice were infected intraperitoneally with 1 × 10^4^ Pru (type II) *Toxoplasma* tachyzoites expressing luciferase. (b, c) Gra6 TN mice and BALB/c mice were directly infected intraperitoneally with 1 × 10^4^ (b) Pru (type II) or (c) CEP (type III) *Toxoplasma* tachyzoites expressing luciferase. At day 3, 5, 7 and 10 post infection parasite load was estimated by luminescence using an *in vivo* imaging system. Each symbol represents one mouse; small horizontal lines indicate the mean, the bottom and top of the box are the first and third quartiles. Data are representative for at least two independent experiments with three to five mice (a, b) or one experiment with three mice (c). Statistical significance was calculated using two‐way analysis of variance**.**

We next aimed to characterize the protective capabilities of the Gra6 TN cells *in vivo*, as well as the response that these mice would show towards direct challenge with *Toxoplasma*. For this we transferred either 1 × 10^6^ WT BALB/c CD8 T cells compared with naive or activated Gra6 TN CD8 T cells into BALB/c recipients and challenged the mice with 1 × 10^4^ *Toxoplasma* Pru intraperitoneally. Gra6 TN CD8 T cells efficiently controlled the parasite load from day 7 post‐infection onwards (Fig. 4a). Previously peptide‐activated Gra6 TN T cells were even more effective at reducing parasite load at 7 days post‐infection (Fig. 4a). Gra6 TN mice directly infected with 1 × 10^4^ *Toxoplasma* Pru exhibited a much‐increased control of parasite load compared with WT BALB/c infected mice (Fig. 4b). Already 5 days post infection, the *Toxoplasma* load in the peritoneum was on average 1·5 orders of magnitude lower than in WT animals (Fig. 4b). Gra6 TN mice directly infected with *Toxoplasma* CEP, a type III strain that does not express the Gra6 epitope HF10, were not able to control parasite burden better than WT mice (Fig. 4c).

## Discussion

We generated L^d^‐restricted TN TCR mice specific to the immunodominant epitope HF10 from the dense granule protein Gra6 in order to obtain a source of these CD8 T cells. As previously described,^8^ the nucleus from a *Toxoplasma*‐specific T‐cell is harvested and transferred into an enucleated mouse oocyte that can then develop into a blastocyst and be used to generate embryonic stem cells. In this process, the antigen‐specific T cells are directly isolated from an infected mouse, which circumvents the necessity of lengthy *in vitro* culture thereby eliminating the skewing of the T‐cell repertoire to adapt to these artificial conditions. The derived embryonic stem cells were then used to generate TN mice that harbour their TCR in the endogenous locus under their natural promoters without any genetic trace. TN mice can be generated with relative ease and are now available for several model and pathogen antigens. Currently the repertoire encompasses B‐cell TN mice and CD8 T‐cell TN mice.^8^, ^24^, ^25^, ^26^, ^27^

We established that the TCR‐*β* chain of our TN mice and a previously described Gra6‐specific hybridoma were almost identical in the CDR3 region responsible for the interaction with the peptide. No discernable homology was found in the TCR‐*α* chain of the TN mouse versus the Gra6‐specific hybridoma. It is possible that to be able to recognize HF10, an unusually longer peptide compared with the typical nonamers presented by L^d^, the CDR3 of the *β*‐chain must be ‘fixed’ or at least display limited sequence variability. It would be interesting to sequence TCRs of other naturally occurring Gra6‐specific clones to test this theory.

Host *Toxoplasma*‐specific CD8 T cells exhibited a broadly similar activation phenotype as donor Gra6 TN T cells in both the mLN and the brain. Specifically, both activated T cells demonstrate low CD62L and high CD69 levels. Most CD8 T cells found in the brain can be assumed to be *Toxoplasma*‐specific, as uninfected brains do not contain CD8 T cells. It is noteworthy that the host Gra6‐specific CD8 population in the mLN as well as the polyclonal *Toxoplasma*‐specific CD8 population in the brain displayed KLRG1^low^ phenotypes. In contrast, donor Gra6 TN CD8 T cells exhibited KLRG1^high^ expression at both sites. The presumably polyclonal response of the host CD8 T cells in the brain to varying *Toxoplasma* epitopes and the polyclonal Gra6‐specific response in the mLN versus the monoclonal response of Gra6 TN CD8 T cells may be responsible for this observed difference. In viral infections, it has been shown that both KLRG1^low^ and KLRG1^high^ perform equally well as effector cells.^28^ In terms of memory potential, high KLRG1 expression on all Gra6 TN CD8 T cells in the mLN and brain suggests that this population is destined for terminal short‐lived effector cells.^29^

We have previously shown that the *Toxoplasma* load in the acute phase of infection can be lowered by adoptive transfer of naive CD8 TN T cells specific for one epitope in the susceptible C57BL/6 mouse model.^8^ Unsurprisingly, in this model we did not observe a significant increase in survival when the mice were challenged with a lethal dose of *Toxoplasma*. Most likely, the transferred T‐cell clone is not the immunodominant one, or alternatively a cocktail of CD8 T‐cell specificities would be needed. In the resistant BALB/c model, we have tested the ability of three different Rop7‐specific TN T cells to lower the *Toxoplasma* load after adoptive transfer and did not observe this capability (EMF and HP, unpublished observations). Again, this is not surprising, as the CD8 T cells specific for Rop7 only arise in the chronic phase of infection and can therefore not represent the immunodominant acute‐phase response.

In contrast, the peptide HF10 from Gra6 has been shown to be the mediator of the immunodominant CD8 T‐cell response in BALB/c mice.^5^ BMDCs pulsed with HF10 peptide elicited a protective immune response in H‐2d B10.D2 mice. Moreover during the acute stage in BALB/c mice, parasites expressing an HF10‐containing Gra6 were cleared faster than parasites expressing a polymorphic version of Gra6, devoid of HF10.^17^ It was therefore plausible to assume that CD8 T cells specific for Gra6 would be able to significantly reduce parasite burden. Indeed, when we transferred Gra6 TN T cells into BALB/c mice and subsequently challenged them with *Toxoplasma*, we could observe that naive Gra6 TN cells could reduce the parasite burden. This protective effect is completely ablated when the mice are infected with a *Toxoplasma* strain that does not harbour the Gra6 epitope. When employing *in vitro* peptide‐activated Gra6 TN T cells, we observed an even more efficient reduction in parasite burden.

In this study, we have generated a fully functional TN CD8 T‐cell mouse specific for the immunodominant *Toxoplasma* epitope Gra6 on a BALB/c background. We confirmed that Gra6‐specific TN CD8 T cells are able to productively lower parasite burden during the acute phase of *Toxoplasma* infection in a BALB/c mouse. Importantly, the Gra6 TN mice as well as our previously generated Rop7‐specific ones are on a BALB/c background, a mouse strain that readily progresses into the chronic phase of *Toxoplasma* infection, in striking contrast to C57BL/6 models that generally succumb during the acute phase. This mouse model can provide a large number of non‐activated CD8 T cells specific for the *Toxoplasma* immunodominant epitope, with one affinity for the MHC I loaded with the Gra6 peptide. Properties of the acute phase of infection CD8 T cells can therefore be studied and further inform on determinants that are important for vaccine development. Additional studies centred around the properties of CD8 T cells in the clinically important chronic phase of *Toxoplasma* infection will be facilitated by employing both of these BALB/c models of TN CD8 T‐cell mice.

## Disclosure

The authors declare no competing interests.

## Supporting information


**Figure S1**. The phenotype of donor cells does not change in the course of infection.
