# Supplementary figures and images for: Transnuclear CD8 T cells specific for the immunodominant epitope Gra6 lower acute‐phase Toxoplasma gondii burden
Source: Immunology. 2016 Aug 17;149(3):270–9. doi: 10.1111/imm.12643 (PMC5046057; doi:10.1111/imm.12643)

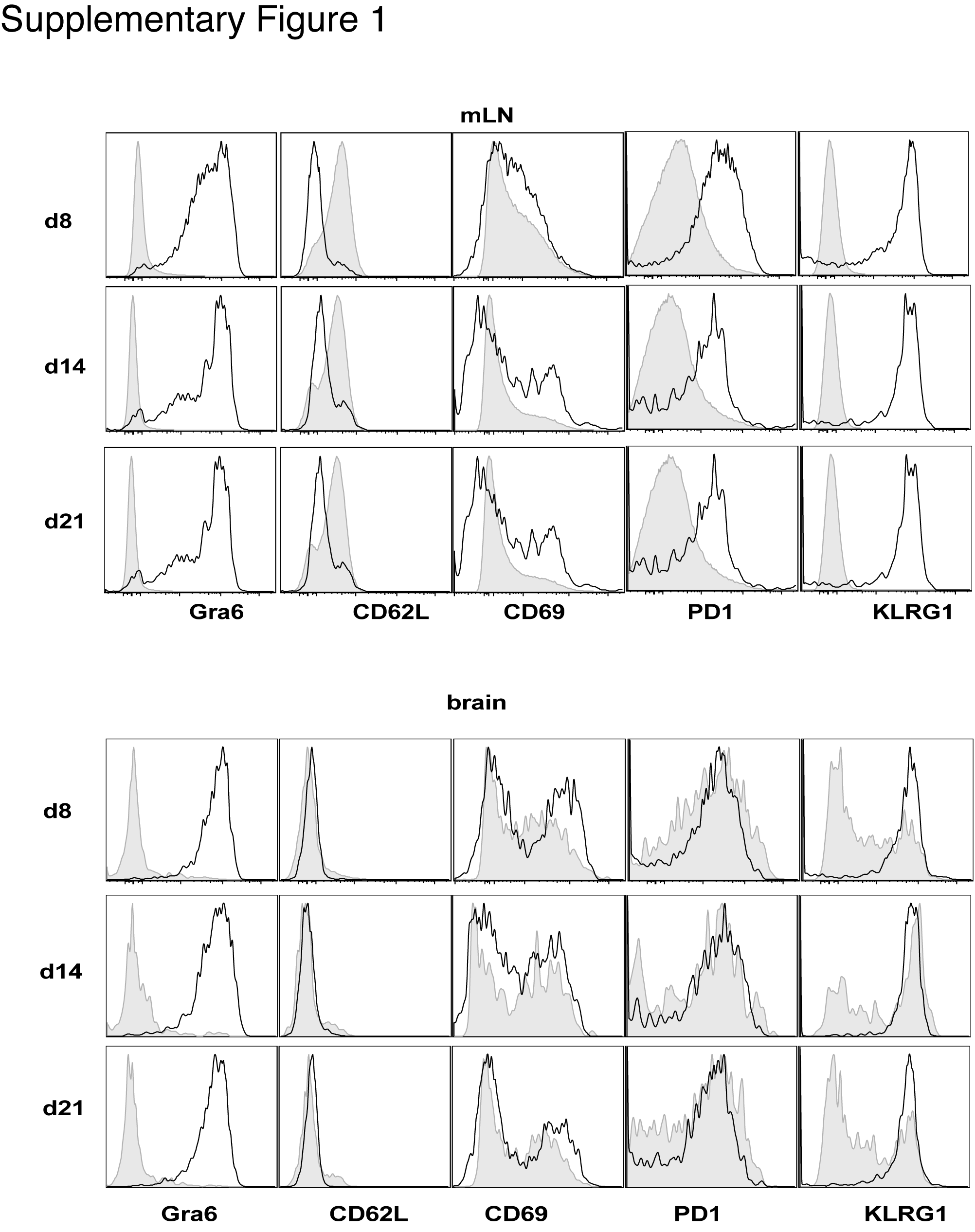

Supplement: Supplementary file 1 — Figure S1. The phenotype of donor cells does not change in the course of infection. [file IMM-149-270-s001.tif]
