# Supplementary material for: Transnuclear CD8 T cells specific for the immunodominant epitope Gra6 lower acute‐phase Toxoplasma gondii burden
Source: Immunology. 2016 Aug 17;149(3):270–9. doi: 10.1111/imm.12643 (PMC5046057; doi:10.1111/imm.12643)
Supplement: Supplementary file 2 [file IMM-149-270-s002.docx]

**Supplementary Information**

**Supplementary Figure 1. The phenotype of donor cells does not change in the course of infection.**

Phenotype of Gra6-specific host and adoptively transferred Gra6 TN cells at day 14, and day 21 post-infection in the mesenteric lymph nodes and brain of *Toxoplasma* infected BALB/c recipient mice. Donor cells were gated as live, CD3^+^ CD8^+^ and CD90.2^+^ (black line), host cells were gated as live, CD3^+^ CD8^+^ and CD90.2^-^ (grey shaded histogram).
